# A Prospective Study Investigating the Impact of Obesity on the Immune Response to the Quadrivalent Influenza Vaccine in Children and Adolescents

**DOI:** 10.3390/vaccines10050699

**Published:** 2022-04-29

**Authors:** Michelle Clarke, Suja M. Mathew, Lynne C. Giles, Alexia S. Pena, Ian G. Barr, Peter C. Richmond, Helen S. Marshall

**Affiliations:** 1 Women’s and Children’s Health Network, North Adelaide, SA 5006, Australia; michelle.clarke@adelaide.edu.au (M.C.); suja.mathew@adelaide.edu.au (S.M.M.); alexia.pena@adelaide.edu.au (A.S.P.); 2 The Robinson Research Institute and Adelaide Medical School, The University of Adelaide, Adelaide, SA 5005, Australia; 3 School of Public Health and The Robinson Research Institute, The University of Adelaide, Adelaide, SA 5005, Australia; lynne.giles@adelaide.edu.au; 4 WHO Collaborating Centre for Reference and Research on Influenza, Melbourne, VIC 3000, Australia; ian.barr@influenzacentre.org; 5 Discipline of Pediatrics, University of Western Australia, Perth, WA 6009, Australia; peter.richmond@uwa.edu.au; 6 Wesfarmers Centre of Vaccines and Infectious Diseases, Telethon Kids Institute and Perth Children’s Hospital, Perth, WA 6009, Australia

**Keywords:** influenza, vaccination, obesity, children, adolescents

## Abstract

Obesity can increase the severity of influenza infection. Data are limited regarding immune responses to influenza vaccination in obese children. We aimed to investigate the impact of obesity on quadrivalent influenza vaccine responses in children. Children with obesity (body mass index (BMI) ≥ 95th percentile for age and gender) and children without obesity (BMI < 95th percentile) were enrolled in the study. Blood samples were collected before, 1, and 6 months after influenza vaccination, to measure antibody responses by haemagglutination inhibition (HI) assay. Vaccine immunogenicity outcomes were compared between children with and without obesity. Forty-four children (mean age 13.3 ± 2.1 years, 18 males and 14 with obesity) completed the 6-month study. More than 90% of the participants with and without obesity had seroprotective antibody titres (HI ≥ 40) at both 1 and 6 months following vaccination for each of the four influenza strains (A/H3N2, A/H1N1, B/(Victoria) and B/(Yamagata)). Influenza-specific geometric mean titres at baseline, 1, and 6 months post-vaccination were similar between children with and without obesity for all influenza vaccine strains. Children with and without obesity have robust, sustained antibody responses over 6 months to the quadrivalent influenza vaccine.

## 1. Introduction

Worldwide, approximately 650 million adults and 340 million children and adolescents are estimated to have obesity [1]. In Australia, more than 25% of children and adolescents are classified as either overweight or obese [2]. Persons with obesity are at increased risk of severe outcomes from influenza infection [3,4]. A number of studies including adults and children have demonstrated that obesity and/or morbid obesity are risk factors associated with influenza hospitalisation [4,5,6,7] and intensive care admission [3,4].

Influenza is an infectious respiratory virus that can affect people of all ages and can cause illnesses of varying severity. In Australia, influenza viruses usually circulate from April–October but infections may occur all year round [8]. Currently, many countries including Australia recommend influenza vaccines to protect individuals from influenza. Expert advisory groups in both Australia and the United States specifically identify individuals with obesity/morbid obesity amongst population groups for whom influenza vaccination is highly recommended, due to their increased risk of severe complications from influenza infection [8,9]. The association between obesity and increased severity of respiratory infections became apparent during the 2009 H1N1 pandemic and again during the COVID-19 pandemic and was attributed to various factors including defective cellular responses, systemic inflammation, and altered hormones including reduced adiponectin and increased leptin. These various physiological changes associated with obesity may play a role in both the ability to effectively manage influenza virus infection and develop robust vaccine immunogenicity [10,11,12,13].

Influenza vaccines currently used in Australia are either split virion or subunit inactivated quadrivalent vaccines and are available for anyone over the age of 6 months. An inactivated influenza vaccine containing an adjuvant is also available for individuals over 65 years of age. Vaccine protection is expected to last for the whole season but is considered optimal during the first 3–4 months after vaccination [8]. Whilst vaccine effectiveness for the prevention of influenza may vary by year and by influenza strain [14], the impact that obesity has on the response to influenza vaccination is unclear, with conflicting findings from various studies and reviews [12,13,15,16]. A prospective observational study following more than 1000 adults vaccinated with a trivalent influenza vaccine found that, compared with vaccinated healthy-weight adults, vaccinated adults who were obese were twice as likely to develop confirmed influenza or influenza-like illness [12]. Another study suggested improved or equivalent immunogenicity at 1-month post-influenza vaccination for obese vs. non-obese adults, but a greater decline in antibodies at 12 months for obese vs. non-obese adults [15]. There are limited data on influenza vaccine immunogenicity responses in obese children [17,18,19]. This study is the first to characterize the development and persistence of influenza-specific antibodies through to 6 months following quadrivalent influenza vaccine administration in obese children and adolescents compared to their non-obese peers.

The aim of this study was to investigate the impact of obesity on the immune response to the quadrivalent influenza vaccine at 1 and 6 months post-vaccination for children and adolescents aged 9–18 years.

## 2. Materials and Methods

### 2.1. Study Participants and Procedures

This prospective cohort study was conducted at the Women’s and Children’s Hospital in Adelaide, South Australia during 2019–2020. Eligible participants were aged between 9 and 18 years and provided written informed parental consent and participant assent. Exclusion criteria included any contraindication to administration of quadrivalent influenza vaccine, receipt of influenza vaccination within the previous 6 months or any chronic medical condition or syndromal/immunosuppressive disorder. Previous influenza vaccination more than a year ago was not considered an exclusion criterion. Participants were enrolled through adolescent obesity and paediatric outpatient clinics, as well as through invitations sent to an in-house research database. Participants’ height, weight and waist circumferences were measured by a study investigator at the first study visit. An online, paediatric BMI Z-score calculator based on the Centres for Disease Control and Prevention (CDC) growth charts was used to determine the participant’s body mass index (BMI), BMI Z-scores, and percentiles [20]. Participants were classified as either having obesity or not, with obesity being defined as a BMI greater than or equal to the 95th percentile for age and gender.

### 2.2. Influenza Vaccine and Vaccination

Licensed seasonal quadrivalent (FluQuadri^®^, Sanofi, New York, PA, USA) influenza vaccines were administered to all enrolled participants. Vaccine composition for both 2019 and 2020 contained antigens from the four recommended influenza strains: A/H3N2, A/H1N1, B/(Victoria), and B/(Yamagata). For A/H3N2, the FluQuadri^®^, vaccine composition included antigens from A/Switzerland/8060/2017 (H3N2)-like virus in 2019 and A/South Australia/34/2019 (H3N2)-like virus in 2020. For A/H1N1, the FluQuadri^®^, vaccine composition included antigens from A/Michigan/45/2015 (H1N1)pdm09-like virus (2019) and A/Brisbane/02/2018 (H1N1)pdm09-like virus (2020). For B/(Victoria) lineage strains, antigens from a B/Colorado/06/2017-like virus (B/Victoria lineage) were used in the 2019 formulation and B/Washington/02/2019-like virus antigens were included in the 2020 vaccine. For B/(Yamagata), antigens from a B/Phuket/3073/2013-like virus (B/Yamagata lineage) were used for both the 2019 and 2020 formulation Vaccines were administered according to the Australian Immunisation handbook recommendations by trained immunisation nurses or study doctors [8].

### 2.3. Serology and Assessment of Immunogenicity

A blood sample was collected immediately prior to the administration of the influenza vaccine to measure baseline influenza-specific antibody titres at visit 1. Further study visits were scheduled for approximately 1 month and 6 months after vaccination for collection of additional blood samples to measure post-vaccination influenza antibody titres. Serum samples were stored frozen and sent to the WHO Collaborating Centre for Reference and Research on influenza (Melbourne) upon study completion to measure antibody titres by haemagglutination inhibition (HI) assay according to the WHO method [21]. Viruses used for HI analysis were egg isolates matched to the vaccine composition. For all assays, viruses were used at a concentration of 4HA units/25uL. Turkey red blood cells (1% (*v*/*v*)) were used in all HI assays. Antibody titrations were performed in duplicate with pre-vaccination, 1-month post-vaccination, and 6-month post-vaccination samples titrated simultaneously on the same day. HI results that were negative (i.e., agglutination apparent at 1:10 dilution) were assigned a titre of 5 for subsequent calculations. Clinical immunogenicity outcomes were based on the ’Note for guidance on harmonization of requirements for influenza vaccines’ published by the European Agency for the Evaluation of Medicinal Products (EMA), Committee for Proprietary Medicinal Products (CPMP) [22]. These included assessment of seroprotection (SP), defined as HI titres ≥ 1:40, and seroconversion (SC), defined as either (i) a pre-existing antibody HI titre of 1:10 or less at baseline and post-vaccination titre of 1:40 or more at 1 month (21–45 days) or (ii) a four-fold or greater rise in titre between day baseline and 1-month post-vaccination where pre-existing antibody level exceeded 1:10. Geometric means of reciprocal HI titres at each time-point and geometric mean titre ratios (GMTR) were also calculated and compared against CPMP criteria for expected mean fold increases. The proportion of participants achieving a higher threshold (HI titres ≥ 1:110) was also calculated and compared between groups.

### 2.4. Statistical Analysis

Descriptive statistics were derived for participants’ demographic and BMI characteristics for the children with and without obesity (means and standard deviations (SD) or number (%), as appropriate). The percentage of participants achieving seroprotective HI titres and seroconversion post-vaccination were derived for the two groups for each of the H3N2, H1N1, B(Yamagata) and B(Victoria) strains, and Chi-squared tests of association, or Fisher’s exact tests, as appropriate, were used to compare between groups. Geometric mean titres (GMT) and 95% confidence intervals (CI) were calculated at each time-point, and the GMTR ratios and 95% CIs were also calculated for 1-month and 6-months post-vaccination for each of the four strains. GMTs and GMTRs at each time-point were compared for the obese and non-obese groups using Mann–Whitney tests. Statistical significance was assessed at the 0.05 level and Stata version 17.0 was used in all analyses.

### 2.5. Ethics Approval and Study Registration

This study was approved by the Women’s and Children’s Health Network Human Research Ethics Committee (HREC/17/WCHN/83) and registered in the Australian and New Zealand Clinical Trials Registry (ACTRN12617001086358) prior to commencement of recruitment.

## 3. Results

**Figure 1:**
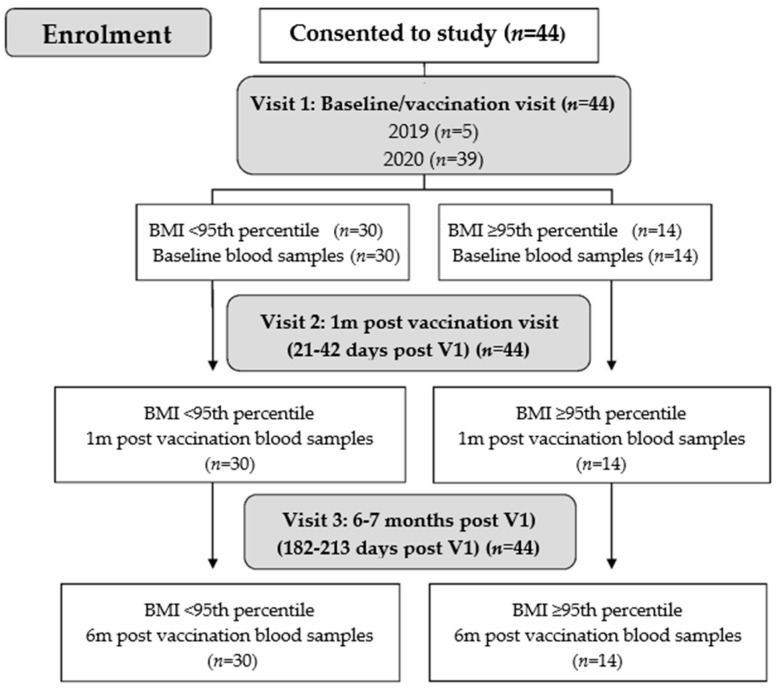
Flowchart of participants and study design.

All study participants received the quadrivalent influenza vaccine (FluQuadri, Sanofi-Aventis, New York, PA, USA) and had blood samples collected at each time-point (Figure 1).

### 3.1. Characteristics of Study Participants

The study involved a total of 44 participants including 26 females (59%) and 18 males (41%). The mean age was 13.3 years (SD: 2.3, range 9–17 years). The BMI Z-scores ranged from −2.12 to 2.89. Fourteen participants (32%) had obesity (BMI ≥ 95 percentile) and 30 were non-obese (BMI < 95 percentile). Of the 30 non-obese participants, the majority (24/30, 80%) had a BMI within the healthy weight range (5–85th percentile). Five participants had a BMI within the 85–95th percentile (overweight) and one participant had a BMI <5th percentile (underweight). Participants with obesity had a mean BMI Z-score of 2.27 (SD 0.28) and participants who were not obese had a mean BMI Z-score of 0.12 (SD 0.80). Almost half of the participants (20/44, 45%) had received an influenza vaccine in the previous 3 years. Characteristics of the obese and non-obese groups were similar (Table 1).

### 3.2. Vaccine Immunogenicity

#### 3.2.1. Geometric Mean Titres and Geometric Mean Titre Ratios

**Figure 2:**
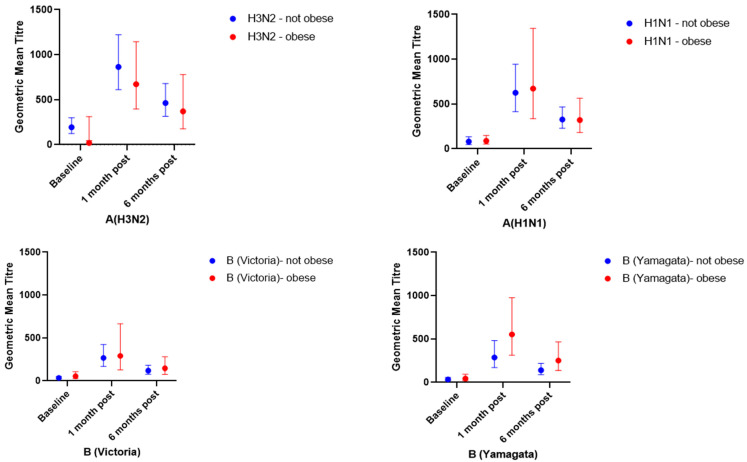
GMT (95% CI) by obesity category and time-point for each strain included in the quadrivalent influenza vaccine.

Both obese and non-obese groups showed a robust increase in GMT for each of the influenza strains (H3N2, H1N1, B/Victoria, B/Yamagata) at 1-month post-influenza vaccination (Table 2/Figure 2), and the GMTs were similar between the groups for all four influenza strains. At 6-months post-influenza vaccination, GMTs remained well above pre-vaccination levels and again were similar between the obese and non-obese groups for all four influenza strains. The GMTRs for each of the 1-month post-baseline and the 6-month post-baseline were also similar for the obese and non-obese groups for all four influenza strains. The 6-month GMTR for H3N2 was 2.4 (95% CI 1.7–3.4) for the non-obese group compared to 3.1 (1.8–5.5) for the obese group. For the remaining influenza strains, the 6-month GMTRs were similar for both the obese and non-obese groups and exceeded 2.5.

#### 3.2.2. Seroconversion and Seroprotection

More than 60% of obese and non-obese participants demonstrated seroconversion at 1-month post-influenza vaccination for all four influenza strains. Both groups exceeded the CPMP criteria of >40% seroconversions for all four influenza strains, demonstrating robust vaccine responses in the study participants, regardless of obesity status (Table 2).

More than 90% of non-obese and obese participants demonstrated seroprotective titres ≥ 40 at both 1- and 6-months post-influenza vaccination for all four influenza strains (Table 2). However, it is important to note that a high proportion of participants had seroprotective titres for influenza A strains (40/44, 91% for H3N2 and 36/44, 82% for H1N1) at baseline (prior to receiving influenza vaccination), reflecting immunity from either prior influenza vaccination or prior influenza infection. The percentage demonstrating seroprotective titres for B/Victoria increased from 55% (24/44) at baseline to 95% (42/44) at 1-month post-vaccination and remained high at 93% (41/44) 6-months post-vaccination. For B/Yamagata, the percentage demonstrating seroprotective titres at baseline was also 55% (24/44) at baseline, 95% (42/44) at 1-month post-vaccination and persisted at 95% (42/44) at 6-months post-vaccination (Table 2).

**Figure 3:**
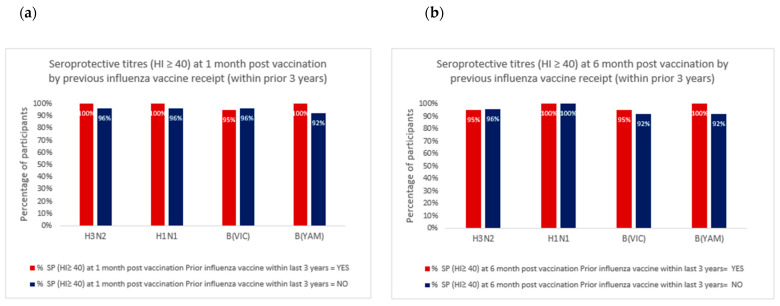
Percentage of participants with seroprotection (≥40) HI titres at (**a**) 1 month and (**b**) 6 months post-influenza vaccination by recent previous influenza vaccination.

All of the participants with obesity demonstrated seroprotective titres for all four influenza strains at 1-month post-vaccination. Six participants in the non-obese group (BMI range in 36–86th percentile) did not demonstrate seroprotective titres to one influenza strain at 1-month post-vaccination. Of these, two participants failed to demonstrate seroprotective titres for B/Victoria, two participants failed to demonstrate seroprotective titres for B/Yamagata, one participant did not achieve seroprotective titres for H1N1, and one participant did not achieve seroprotective titres for H3N2 at 1 month following influenza vaccination (Table 2). The proportion of participants with seroprotective titres was similar for those who had and had not received a previous influenza vaccination (within the prior 3 years) (Figure 3).

Using a higher HI titre threshold of 1:110 to evaluate immunogenicity between groups, the results remained similar between children and adolescents with and without obesity. At 1-month post-vaccination, more than 95% of children and adolescents without obesity demonstrated HI titres > 110 for H3N2 and H1N1 compared with 100% of children and adolescents with obesity. For B/Victoria and B/Yamagata, more than 75% of children with and without obesity demonstrated HI titres > 110 (Table 2).

## 4. Discussion

This study demonstrated that obesity did not impair vaccine responses and that adequate vaccine responses were observed across both obese and non-obese groups for all four influenza strains studied. The study results confirmed robust GMT and GMTR at 1- and 6-months following vaccination, with more than 60% of participants achieving seroconversion at 1-month post-vaccination and 90% demonstrating seroprotective titres (HI ≥ 40) at both 1 and 6 months following vaccination.

Criteria for the assessment of influenza vaccine immunogenicity were developed and published by the EMA CPMP [22]. These guidelines provide serological analyses and expected performance criteria for influenza vaccines for adults between 18–60 years of age. Serological assessments are performed on blood samples collected at approximately 3 weeks after vaccination and include the mean fold increase between baseline and post-vaccination HI titre of at least 2.5, with more than 40% of participants demonstrating seroconversion and/or greater than 70% participants achieving HI titres ≥ 40. Results from our study demonstrated that for children and adolescents with and without obesity, all immunogenicity analyses met or exceeded these CPMP/EMA criteria for evaluation of influenza vaccines at 1-month post-vaccination. Whilst there is no guidance for serological immunogenicity expectations at 6-months post-vaccination, all CPMP thresholds/criteria continued to be met for both seroconversion and seroprotection to all influenza strains for both obese and non-obese groups at 6-months post-vaccination. Additionally, GMT remained at least twice that of baseline for all influenza strains for both obese and non-obese participant groups. Thus, the results from this study suggest robust, persistent immune responses through to at least 6-months post-quadrivalent influenza vaccination for children and adolescents, with and without obesity.

Whilst the CPMP guidelines are based on influenza vaccine responses for adults, one study of the protective correlate in more than 750 children aged 6–17 years found that a HI titre of ≥ 40 conferred approximately 50% protective efficacy [23] and was therefore similar to that of adults. However, it was suggested that a higher titre (1:110) may be more appropriate for children [24]. Using this higher titre as a threshold for protection did not alter the study findings, with more than 95% of obese and non-obese participants demonstrating titres greater than or equal to 110 for both H3N2 and H1N1 and more than 75% of participants with and without obesity meeting this threshold for B/Victoria and B/Yamagata HI titres (Table 2).

There are an increasing number of recent studies assessing the association between obesity and influenza vaccine responses in adults with conflicting findings [25,26,27]. In contrast, there are only a few small studies describing influenza vaccine responses in obese children and adolescents [17,18,19], and none of these studies characterizes the immune responses through to 6-months post-vaccination. One study, which included a cohort of obese children (*n* = 13) and obese adolescents (*n* = 21), assessed antibody responses following monovalent influenza vaccination and found no significant differences in seroconversion or seroprotection by weight category (underweight, normal weight, overweight and obese) after one or two doses [17]. Another study assessed the impact of obesity on vaccine responses in 28 overweight/obese and 23 normal-weight children aged 3–14 years in Italy. These authors found similar or improved antibody responses at 4-weeks and 4-months post-trivalent influenza vaccination in overweight/obese children when compared to normal-weight children [18]. A third study evaluated the impact of vitamin D and BMI on antibody titres at day 21 after either live, attenuated, or inactivated influenza vaccine (IIV) in children aged 3–17 years [19]. That study included 52 children (17 with BMI ≥ 95th percentile) who received IIV and 83 (29 with BMI ≥95th percentile) who received a live, attenuated influenza vaccine (LAIV) and compared results with children and adolescents with a BMI <95th percentile. The authors concluded that for IIV there was no significant association between BMI and antibody titres for A/H1N1 or either B strain, however, a reduction in day 21 log antibody titres was observed for A/H3N2 in obese children (BMI ≥ 95th percentile) compared to non-obese children (BMI < 95th percentile).

The data from our present study and each of these small observational studies suggest that there is no apparent impairment in overall influenza vaccine antibody responses for children with obesity.

Strengths of our study include detailed immunogenicity data for all four influenza strains used in recent influenza vaccines for obese and non-obese children and adolescents. Furthermore, our study provides information on the persistence of these antibody responses up to 6 months following vaccination, which is the typical length of an influenza season in Australia. Another strength of the study is the completeness of the data collected. However, this study is limited by its small sample size and therefore, small but clinically important differences between obese and non-obese groups may be difficult to detect. The participants may not represent the wider population of children and adolescents, although this limitation also applies to other studies in this area. Our study population also included a high proportion of participants with seroprotective HI titres at baseline, particularly to influenza strains H1 and H3, which may influence the likelihood of seroprotection at 1 month and the persistence of antibodies at 6 months. Whilst we excluded participants who had received an influenza vaccine in the previous 6 months, participants who had received an influenza vaccine prior to 6 months or those who had a previous influenza-like illness were not excluded. The time taken for vaccine-induced antibodies to decay to one-half of the post-vaccination titre for children receiving an inactivated influenza vaccine was estimated at approximately 4 months for H1N1 and 8–9 months for H3N2 [28]. However, prior natural influenza infection is likely to induce a longer duration of antibody persistence than previous vaccination with inactivated influenza vaccines [29,30]. It is possible that this may have contributed to the higher than expected proportions with seroprotective antibody titres at baseline. Whilst results may differ for a highly seronegative population, in our study, restricting analyses to those participants who had HI titres < 40 at baseline did not alter findings with similar results for children and adolescents with and without obesity. Prior vaccination or infection may potentially influence subsequent vaccine responses [31] and may provide cross-protective immunity against future novel influenza virus exposure [32]. Our study did not examine cross-protection against non-vaccine strains, however, we did compare seroprotection between participants who had (*n* = 20) and had not (*n* = 24) received a prior recent influenza vaccine and found similarly high levels of seroprotection.

A larger sample size would allow us to explore other potential confounders of the sufficiency of vaccine responses in children such as sex and prior vaccination. Furthermore, our study did not investigate innate host responses such as cytokine and T cell population/proliferation, which may also provide additional valuable insights for comparing vaccine responses between children and adolescents with and without obesity. Finally, it is important to note that equivalent HI responses may not infer equivalent protection against influenza for groups with and without obesity. A prospective observational study involving more than 1000 adult participants who received trivalent influenza vaccine showed that, compared with vaccinated healthy weight participants, vaccinated obese participants had twice the risk of influenza or influenza-like illness, despite similar immunogenicity [12].

## 5. Conclusions

This study provides encouraging results to support sustained immune responses in both obese and non-obese children and adolescents following a single dose of the quadrivalent influenza vaccine. The maintenance of high vaccine immunogenicity at 6-months post-vaccination in both obese and non-obese children and adolescents is encouraging, however, further research is needed to ensure that influenza vaccine efficacy is adequate in protecting children with obesity from severe influenza disease

## Figures and Tables

**Table 1:** Characteristics of study participants by BMI category.

	Not Obese (*n* = 30) (BMI < 95th Percentile)	Obese (*n* = 14) (BMI ≥ 95th Percentile)
Age: mean years (SD)	13.3 (2.3)	13.4 (1.9)
Gender: % male	13/30 (43%)	5/14 (36%)
Prior influenza vaccine (during last 3 years)	13/30 (43%)	7/14 (50%)
Height (cm): min, max, mean (SD)	135–182, 161 (13)	144–179, 163 (11)
Weight (kg): min, max, mean (SD)	29–76, 52 (13)	51–139, 90 (23)
BMI percentile: min, max, median, (IQR)	1.7, 92.6, 48 (36–80)	97.0, 99.8, 99 (98–99)

**Table 2:** Influenza vaccine immunogenicity by influenza strain and obesity status.

	H3N2			H1N1			B(Victoria)			B(Yamagata)		
	Not Obese (BMI < 95th Percentile) (*n* = 30)	Obese (BMI ≥ 95th Percentile) (*n* = 14)	*p* Value	Not Obese (BMI < 95th Percentile) (*n* = 30)	Obese (BMI ≥ 95th Percentile) (*n* = 14)	*p* Value	Not Obese (BMI < 95th Percentile) (*n* = 30)	Obese (BMI ≥ 95th Percentile) (*n* = 14)	*p* Value	Not Obese (BMI < 95th Percentile) (*n* = 30)	Obese (BMI ≥ 95th Percentile) (*n* = 14)	*p* Value
Baseline GMT (95% CI)	193	119	0.30	80	88	0.91	34	54	0.24	34	44	0.56
	(124–299)	(45–312)		(48–134)	(53–148)		(23–51)	(27–108)		(20–57)	(21–94)	
1 month post vaccination GMT (95% CI)	864	672	0.35	625	672	0.96	266	289	0.91	285	552	0.15
	(612–1220)	(395–1144)		(415–943)	(336–1344)		(168–422)	(127–664)		(169–481)	(312–975)	
6 months post vaccination GMT (95% CI)	463	371	0.66	327	320	0.82	118	145	0.66	139	250	0.14
	(316–679)	(177–778)		(229–468)	(182–564)		(78–180)	(75–281)		(89–218)	(137–455)	
GMTR (95% CI) (1 m/baseline)	4.5	5.7	0.50	7.8	7.6	0.80	7.8	5.4	0.08	8.4	12.5	0.39
	(3.1–6.6)	(2.9–11.0)		(4.6–13.2)	(2.8–20.4)		(5.4–11.4)	(2.9–10.0)		(5.1–13.7)	(5.0–31.0)	
GMTR (95% CI) (6 m/baseline)	2.4	3.1	0.36	4.1	3.6	0.67	3.5	2.7	0.35	4.1	5.7	0.48
	(1.7–3.4)	(1.8–5.5)		(2.6–6.5)	(1.5–8.5)		(2.5–4.8)	(1.6–4.5)		(2.8–6.0)	(2.5–12.9)	
% seroconversion ^1^ 1 m post	18/30 (60%)	9/14 (64%)	1.00	21/30 (70%)	9/14 (64%)	0.74	27/30 (90%)	10/14 (71%)	0.18	23/30 (77%)	10/14 (71%)	0.72
% seroprotection ^2^ (HI ≥ 40)												
baseline	28/30 (93%)	12/14 (86%)	0.58	23/30 (77%)	13/14 (93%)	0.40	14/30 (47%)	10/14 (71%)	0.20	16/30 (53%)	8/14 (57%)	1.00
1 month post	29/30 (97%)	14/14 (100%)	1.00	29/30 (97%)	14/14 (100%)	1.00	28/30 (93%)	14/14 (100%)	1.00	28/30 (93%)	14/14 (100%)	1.00
6 months post	29/30 (97%)	13/14 (93%)	0.54	30/30 (100%)	14/14 (100%)	1.00	27/30 (90%)	14/14 (100%)	0.54	28/30 (93%)	14/14 (100%)	1.00
% seroprotection ^2^ (HI ≥ 110)												
baseline	23/30 (93%)	7/14 (50%)	0.10	16/30 (53%)	6/14 (43%)	0.75	6/30 (20%)	3/14 (21%)	1.00	7/30 (23%)	4/14 (29%)	0.72
1 month post	29/30 (97%)	14/14 (100%)	1.00	26/30 (97%)	14/14 (100%)	1.00	26/30 (80%)	11/14 (78%)	1.00	23/30 (77%)	13/14 (93%)	0.40
6 months post	29/30 (97%)	11/14 (78%)	0.09	25/30 (83%)	12/14 (86%)	1.00	15/30 (50%)	6/14 (43%)	0.75	19/30 (63%)	10/14 (71%)	0.74

^1^ Seroconversion defined as a four-fold increase in HI titre between baseline and 1-month post-vaccination ^2^ Seroprotection defined as a HI titre ≥ specified threshold at specified time-point. *p*-values are derived from either Mann–Whitney tests (GMT/GMTR) or Fisher exact tests (seroconversion/seroprotection).

## Data Availability

De-identified data presented in this study are available on request from the corresponding author.
